# Reactive Materials in the Removal of Phosphorus Compounds from Wastewater—A Review

**DOI:** 10.3390/ma13153377

**Published:** 2020-07-30

**Authors:** Sylwia Gubernat, Adam Masłoń, Joanna Czarnota, Piotr Koszelnik

**Affiliations:** 1 Doctoral School of Engineering and Technical Sciences, Rzeszow University of Technology, Powstańców Warszawy 6, 35-959 Rzeszów, Poland; d512@stud.prz.edu.pl; 2 Inżynieria Rzeszów S.A., ul. Podkarpacka 59a, 35-082 Rzeszów, Poland; 3 Department of Environmental Engineering and Chemistry, Rzeszow University of Technology, Powstańców Warszawy 6, 35-959 Rzeszów, Poland; askalucz@prz.edu.pl (J.C.); pkoszel@prz.edu.pl (P.K.)

**Keywords:** reactive materials, adsorption, phosphorus, wastewater treatment

## Abstract

Modern technologies designed to treat wastewater containing phosphorus compounds are based on the processes of adsorption and precipitation. In addition, more environmentally friendly and cheaper materials are being sought to ensure greater conformity with overarching assumptions of green chemistry and sustainable development. Against that background, this paper offers a review and analysis of available information on the considered reactive materials that have the capacity to remove phosphorus from wastewater. These materials are categorised as natural (with a sub-division in line with the dominant sorption groups of Al/Fe or Ca/Mg), waste, or man-made. Notably, most studies on sorbents have been carried out in laboratory systems via experimentation under static conditions. Among the natural materials, opoka has the highest sorption capacity of 181.20 g P/kg, while red mud (in the waste material category) is most efficient at binding phosphorus with a level of 345.02 g P/kg. Finally, among the group of commercial materials, *Rockfos*^®^ has the highest sorption capacity of 256.40 g P/kg. In addition, this paper recognises the effect of composition, pH, and physical properties on a reactive material’s capacity to absorb phosphorus, as well as the possibility for further potential use in the production of fertilisers.

## 1. Introduction

Phosphorus is a major biogenic element and one that is essential in the synthesis of protein molecules; it is also a general ingredient crucial to the ongoing processes in living plant and animal cells. Phosphorus is present in compounds such as adenosine triphosphate (ATP), which transfers energy at the molecular level [1]. From a human perspective, this element is extracted from the ground where inorganic orthophosphate deposits are present. Currently, there are no synthetic substitutes for this element. Among the various biogenic compounds, phosphorus stands out with its sedimentation type cycle, lack of a gaseous phase, and impeded transfer in the environment [2]. Where circumstances are alkaline, phosphorus participates via certain sparingly soluble compounds. Only in an acidic environment does dissolution occur.

Civilisational development and population growth have combined to ensure that phosphorus is increasingly considered a strategic resource. About 82% of phosphorus obtained is presently used in agriculture, while 7% is used in the manufacture of animal feed, with the remaining 11% used in pharmaceuticals and medicine or in the manufacture of detergents [3,4].

Given its status as a key component of agricultural fertilisers, phosphorus is a major agent of the eutrophication process wherein organic matter is generated in excessive amounts as bodies of water become over-enriched in nutrients. The consequence is a degradation of natural waters [5]. This explains the desire to remove phosphorus from wastewater to the maximum reasonable (or possible) extent, with recovery of the substance where possible. Large amounts of phosphorus are “lost” to bodies of water, landfills, and wastewater treatment plants [6,7], yet control of the eutrophication process is crucial for ensuring the continuity of aquatic ecosystems in a natural and healthy state. This is vital if people are to be guaranteed drinking water of adequate or desirable quality. To provide adequate protection against eutrophication, wastewater treatment plants have steep requirements for the highest allowable phosphorus concentrations or the minimum percentage of its reduction when discharging treated sewage into water or the ground. In Poland, depending on the Population Equivalent (P.E.: a number expressing the ratio of the sum of the pollution load in wastewater to the individual pollution load in household sewage produced by one person in the same time. In Poland, the BOD5 load from 1 person is assumed to be equal to 60 g O2 per 24 h), the value of the phosphorus concentration in treated sewage cannot exceed 5 mg P/L at PE < 2000, 2 mg P/L at P.E. in a range of 2000–99,999, or 1 mg P/L at P.E > 100,000. The minimum percentage of phosphorus reduction is 80% at P.E. > 10,000 [8].

At wastewater treatment plants, the main methods for removing P are based on chemical-precipitation technologies involving lime, aluminium, and iron salts, as well as the Enhanced Biological Phosphorus Removal (EBPR) process [3]. The use of biological methods may, in some cases, prove insufficient to prevent the eutrophication process effectively. In contrast, chemical methods are expensive due to the need for reagents. Modern technologies are based on the adsorption and precipitation processes. However, in accordance with the green chemistry and sustainable development (ecogreen) concepts, cheap and environmentally-friendly materials are currently being sought [3,4].

The basic process for reducing the amount of phosphate ions in an aqueous environment entails moving the processes of phosphorus circulation at the sludge–water phase boundary towards solid-phase deposition by means of chemical precipitation or adsorption [9]. The latter process is one where molecules, atoms, or ions are bound on the surface or interface of the physical phase. Ion adsorption is a complex process dependent on many different factors. Adsorption onto the surface of adsorbents depends on the type used, i.e., the functional groups present on the adsorbent’s surface. The process of the “uptake” of ions or molecules through an adsorbent surface can be the result of physical or chemical adsorption, hydrogen bonding, ion-exchange, micro precipitation, or condensation in the adsorbent pores [10,11,12]. Under certain conditions, the adsorption process continues until achieving a dynamic equilibrium between the adsorbate concentration remaining in the solution and that on the inner surface of the sorbent. The equilibrium adsorption parameters at the solid–liquid border are determined by analysing the process under stationary or dynamic conditions. Static experiments consist of determining the concentrations of the initial solution and a solution in equilibrium with the adsorbent, which is obtained by shaking the solution with the adsorbent material. Column experiments consist of passing the solution through a filtration layer that is a sorbing material. Adsorbate separation between the solution and adsorbent is described by the adsorption isotherm equations, which determine the ratio between the adsorbed substance and the equilibrium concentration of the solution. Equilibrium parameters provide an understanding of the nature and mechanism of adsorption, the types of interactions, and a determination of the process at the molecular level using a theoretical equation or empirical value [10,11].

For phosphorus removal from wastewater, the chemical composition of the adsorbent is important. Reactive materials are those able to remove substances selectively by sorption or precipitation. For phosphorus removal from wastewater or other waters, the potential material should contain compounds able to bind phosphorus, like Ca, Mg, Fe, and Al. The mechanism for phosphate adsorption by Fe and Al can occur through the exchange of ligands. However for Ca and Mg, the removal of phosphates is achieved via precipitation of sparingly-soluble tricalcium phosphate or struvite [13]. For this reason, phosphorus-sorbing materials are divided according to the dominant sorption forms: Al/Fe or Ca/Mg. Currently, in technology for wastewater treatment from phosphorus compounds, pure reagents containing calcium, iron, and aluminium salts are used in precipitation methods. Due to the tendency to use natural or waste materials, which has both ecological and economic justifications, precipitation methods are commonly replaced by methods based on the filtration, sorption, and biofiltration processes, in which materials such as *Leca^®^, Polonite^®^, Rockfos^®^, Pollytag^®^, Filtralite^®^*, and *Filtra P* are used. These materials were created from natural materials subjected to appropriate modifications to obtain a product with high phosphorus removal efficiency [3].

This study discusses the selected reactive materials based on their use in the selective removal of phosphorus from wastewater. After recognizing the largest possible number of tested materials in this area, materials with high phosphorus removal efficiency were selected based on their real use in wastewater treatment technologies or the motivation for further research on the materials (e.g., modifications to improve their properties in this aspect). Other materials were not included in the review due to their poor efficiency in phosphorus binding or lack of economic or technical justification for their use.

The aim of the work was to present and discuss individual reactive materials that were divided into three groups: natural materials and their modifications, waste, synthesized materials and their modifications, and “man-made” materials. Due to differences in the methods for phosphorus binding, natural materials were separated by the dominance of their sorption group (Al/Fe and Ca/Mg). The materials were characterized by determining their sorption capacity and phosphorus removal efficiency (results from tests conducted under static and dynamic conditions were included), and the impact of physicochemical parameters and process conditions on their effectiveness was assessed.

## 2. Natural Materials and Their Modifications

In search of appropriate phosphorus adsorbents, the first research was carried out on natural materials, due to their easy availability, constituted the basis for knowledge to focus research on waste materials in the future, which in turn may translate into a reduction of waste emissions.

### 2.1. Materials with a Pronounced Al/Fe Sorption Group

One of the materials used in this way is bauxite, which was analysed as the main source of aluminium in the form of clay sedimentary rock. For bauxite, Drizo et al. [14] demonstrated an adsorption capacity of 0.61 g P/kg, while column tests showed that the material was saturated with 350.00 g P/kg after 70 days. The effect of pH was investigated, and the maximum adsorption values for phosphates were shown to be achieved under slightly acidic conditions (pH 3.2–5.4) (Table 1). Altundogan et al. [15,16] found that the adsorption capacity of thermally activated bauxite (heat-treated at 600 °C) was greater for all tested forms of phosphate than for raw bauxite. This likely reflects an increase in the specific surface area (SSA) from 11.0 m^2^/g (raw) to 86.0 m^2^/g (activated as above). For glycerophosphate, thermal modification increased the efficiency of removal from 39.9 to 90.9% [15,16] (Table 1).

Diatomite was also used in the removal processes for P, with both pH and material modifications found to have an impact on the sorption capacity reported (Table 2). With natural diatomite, this value is 10.20 g P/kg at pH = 4.0. The efficiency of removal is lower at a higher pH (where pH = 8.0, the sorption capacity is of only 1.70 g P/kg) [17]. A ferrihydrite-modified form of diatomite can be prepared when treatment with NaOH is followed by the deposition of ferrihydrite on the crude substance. The specific surface area of the material is again increased in this way from 24.77 to 211.10 m^2^/g. Such a modification raises the maximum sorption capacity to 37.30 g P/kg at pH = 4.0 and 13.60 g P/kg at pH = 8.5 [17].

The research conducted by Xie et al. [18] likewise documented an increased SSA of diatomite following modification with lanthanum from 0.64 to 52.60 m^2^/g. In these circumstances, the removal effectiveness from a solution of 5 mg P/L reached 98.2% at the outset.

Another material studied for its capacity to remove phosphorus compounds in wastewater treatment is akadama clay. Research carried out with this sorbent (1 g of material + 200 mL of solution with an initial concentration of 20 mg P/L, was shaken at a speed of 100 rpm for 0 to 540 min) showed that the natural form of akadama clay has a maximum phosphorus adsorption capacity of 5.88 g P/kg [19]. However, the efficiency increased further to 9.19 g P/kg under H_2_SO_4_ activation. Furthermore, this treated form of akadama clay differs from the natural form in providing more effective removal over a wider pH range (from 3.0 to 6.0). In this case, the presence of competitor anions, especially citrate and carbonate, were shown to negatively impact phosphate adsorption. Table 3 compares the results for natural and modified akadama clay.

Laterite represents yet another alternative phosphorus-sorbing material, whose characteristics are shown in Table 4. Under the Langmuir model, the maximum sorption capacity in this case is 2.73 g P/kg. However, 1.65 g P/kg proved possible, even when the concentration of phosphorus compounds was very low at 0.035 mg P/L, demonstrating that this material could have applications in wastewater treatment [20]. Laterite’s effectiveness at removing P is also greater at higher temperatures [21], and an increase is likewise obtained if laterite is modified by acidification [19].

Shale has also been applied to the removal of phosphorus from wastewater in static experiments by Drizo et al. [14], which obtained a maximum sorption capacity equal to 0.65 g P/kg (compared to 0.75 g P/kg in column tests). Table 5 details these sorption properties. 

Moreover, Cyrus et al. [23,24] achieved a maximum sorption capacity of 0.17 g P/kg, where the initial concentration was 1000 mg P/L compared to 0.50 g P/kg, with an initial concentration only one-tenth as high. Sorption experiments generally reveal lower percentage sorption capacity where P concentrations in solution are progressively higher. Shales of a larger fraction size also offer poorer efficiency in removing phosphorus due to their lower surface area and higher hydraulic conductivity than smaller material particles [23,24]. Likewise, recent column studies confirmed the maximum sorption capacities for material of 0.06 and 0.23 g P/kg, where the initial mg P/L concentrations were 5 and 25, respectively [25]. Further, the amount of porosity (the ratio of the volume of voids inside the material to the volume of the whole body) did not affect the sorption capacity of shale. Nevertheless, sorption materials must have a porous structure adequate for their physicochemical properties. The porosity of the material will determine the degree of the removal of impurities of various sizes and thus allow the sorption process to occur [10].

In the discussed aspect of wastewater treatment from phosphorus compounds, the next proposed sorbent is zeolite. Phosphate sorption by the natural form of this material (the main component is clinoptilolite), proceeds through various mechanisms: ion exchange, physical sorption and chemisorption. The relevant literature (as summarised in Table 6) shows the sorption capacities for this substance to range from 0.46 g P/kg [14] to 2.15 g P/kg [26]. Other work obtained 67.5% phosphorus removal using zeolite, where the contact time was 180 min [27]. Zeolite synthesised from fly ash (ZFA) has also been the subject of analysis, as have versions modified with Ca^2+^ (ZFA–Ca), Fe^3+^ (ZFA–Fe), or Al^3+^ (ZFA–Al) [13]. Experiments run with initial concentrations of 1000 mg P/L revealed a universally high efficiency of removal with zeolite, as Table 6 illustrates. However, where P is present at the low concentrations characteristic of real wastewater, ZFA–Al and ZFA–Fe work best.

Bentonite is another reactive material in which the Al/Fe sorption group prevails. Bentonite’s properties make it suitable for modifications to increase natural sorption capacity. As Table 7 shows, these modifications are able to considerably increase the material’s SSA beyond the 37.10 m^2^/g that characterises the natural substance. Among all the bentonite modifications, the best adsorbent is the lanthanum (III)-modified version, which attains a maximum adsorption capacity of 14.00 g P/kg. The similar value of 12.70 g P/kg obtained for hydroxyAl–modified bentonite demonstrates that bentonite-based adsorbents can be highly efficient in removing P [28,29].

### 2.2. Materials with a Pronounced Ca/Mg Sorption Group

Calcite is a natural material whose composition is dominated by the Ca/Mg sorption group. Research on industrial wastewater containing phosphorus in the form of orthophosphates, which are produce during the production of thin-film-transistor liquid-crystal display (TFT–LCD) screens has yielded a maximum sorption capacity for calcite equal to 13.26 g P/kg, with a pH of 6.5 [30]. Other work likewise confirmed a marked capacity to absorb phosphorus [31,32], especially when the environment is alkaline (pH 7.6–12.0) and where phosphate/adsorbent ratios are high. Moreover, calcite can be regarded as environmentally friendly, given its lack of need for further processing and thus its potential use in conditioning acidic soils [31].

Dolomite has also been used to remove P from wastewater, with a demonstrated sorption capacity of 51.02 g P/kg, where 60 mg P/L is the initial concentration [33]. Experiments using distilled water (initial 0.28 mg P/L), ground water (0.34 mg/L), tap water (0.34 mg P/L), and wastewater (0.56 mg P/L) found respective sorption capacities for dolomite of 0.06, 0.072, 0.05, and 0.05 g P/kg. The maximum sorption capacities determined via the Freundlich and Langmuir models are 0.12 and 0.093 g P/kg, respectively [34]. Table 8 offers a further characterisation of dolomite. Research into the effects of additional factors in the adsorption of phosphorus by dolomite found that slightly more P is removed where the pH and/or temperature are higher. Changes to the structure and pore-size distribution may occur with calcination [35].

Yin et al. [36] used naturally occurring sepiolite, which is rich in calcium (CaO at 22.3%). Experimentation showed that the phosphorus sorption across 5–1000 mg P/L concentrations can be accurately described by the Freundlich equation. The estimated maximum sorption capacity was 32.00 g P/kg, which is rare for a natural material. However, phosphate adsorption is progressively lower when the pH value increases to 6.0 from 3.0 and experiences a sharp decline under alkaline conditions. P-removal here is based on a precipitation process with calcium, as confirmed by Scanning Electron Microscopy–Energy Dispersive Spectroscopy (SEM–EDS) analysis [36].

The industry for mining and processing marble produces large amounts of waste, including a powder containing valuable minerals that can be used in the process for binding P from wastewater. The material in question has been analysed in its natural and calcined forms, and Table 9 presents the results. The sorption capacity of powdered marble can reach 103.20 g P/kg for the natural material and as high as 181.20 g P/kg when calcined at 1000 °C [37]. On the other hand, work using a Continuous Stirred Tank Reactor (CSTR) documented a 17.00 g P/kg sorption capacity for natural marble powder [38]. Research on industrial wastewater (pH = 4.76, initial concentration 1000 mg P/L), using a process involving calcined powdered marble, found a value for the remaining phosphorus equal to only 341.40 mg P/L [37].

Other trials with industrial wastewater (pH = 6.2, initial P concentration 226.34 mg/L) confirm a 99.9% efficient removal by calcined marble, where the original dosed material was 11.00 g P/L [39]. Eljamal et al. [40] obtained a removal efficiency of 93.0% with marble dust and synthetic solutions (5 g material + 100 mL solution at an initial concentration of 100 mg P/L, shaken for 120 h at 120 rpm).

Opoka is another sorbent valued for its ability to absorb phosphorus. Research on opoka has analysed its natural form as well as its form when calcined at various temperatures (Table 10) [41,42,43,44,45,46], with both types tested on synthetic solutions containing phosphorus. Thermal treatment increased the sorption capacity significantly due to the presence of calcium carbonate decomposition products such as calcium oxide and carbon dioxide. The calcined opoka achieved a high efficiency of phosphorus removal (12.30–181.81 g P/kg). However, a relevant problem in real-life wastewater treatment might be the increased pH due to calcination, which can exceed 12.0. The thermal treatment of opoka also has an unfavourable economic impact, though a commercial product called *Polonite*^®^ (a 2.0–6.0 mm opoka fraction calcined at 900 °C) is already used to remove P from both wastewater and water from cultivated fields.

Sands used as a filtration material can also achieve the surface retention of phosphorus. In filter beds, binding mainly occurs via adsorption and precipitation with calcium, aluminium, and iron (Table 11). Where pH values are above 6.0, the method of binding phosphorus is based on physical adsorption on iron and aluminium oxides and precipitation precipitation to form sparingly soluble calcium phosphates. In contrast, where the pH is below 6.0, the precipitation of iron and aluminium phosphates (strengite, variscite) increases as the pH decreases [48]. A possible explanation for the above reactions can be found in research on phosphorus removal in Constructed Wetlands (CWs) [49]. The phosphorus adsorption on sands is controlled by the interactions of the following parameters: redox potential, pH, Fe, Al, and Ca. In an acidic reaction, phosphorus is adsorbed on hydrated Fe and Al oxides and can precipitate as insoluble iron and aluminium phosphates. However, precipitation as insoluble calcium phosphate can occur only at pH values higher than 7.0 [49,50]. This decreasing redox potential can cause the conversion of crystalline Al and Fe to an amorphous form. Amorphous hydrated iron and aluminium oxides have a higher sorption capacity than crystalline oxides due to their greater number of individually coordinated surface hydroxyl ions. The mechanism of phosphorus binding entails the exchange of ligands where phosphate displaces water or hydroxyl from the surface of hydrated iron or aluminium oxides to form monodentate and bi-nuclear complexes in the sphere of hydrated oxide coordination [49]. Because the sand’s ability to absorb phosphorus may depend on its Ca, Al, and Fe content, attempts were made to improve the phosphorus removal efficiency of CWs by enriching the sand bed with calcium or iron. The research results have shown that the use of a substrate with a high content of iron and aluminium is effective only during the first months of operation [50]. Studies have also shown that the addition of reactive Ca (CaO or Ca (OH)_2_) is more effective in improving the adsorption capacity of P than the addition of Al and Fe [41,47,50].

According to Arias et al. [51], the maximum sorption capacities estimated using the Langmuir model do not correspond to or correlate with the actual amounts of P removed in column experiments. Although the Langmuir model is intended to describe adsorption alone, Veith et al. [52] revealed that the equation can describe precipitation reactions whose conditions are defined and isolated appropriately. This confirms that more complex reactions occur in sands [51]. In turn, the research conducted by Vohla et al. [53] showed the efficiency of phosphorus removal at the level of 24.0% obtained along with a sorption capacity of 1.90 g P/kg.

Limestone is another material used to remove phosphorus. Research by Johansson et al. [44] showed limestone to have a phosphorus sorption capacity of 0.25 g P/kg, while Drizo et al. [14] gave a value of 0.68 g P/kg. The highest observed value was 1.09 g P/kg, obtained by Bellier et al. [54] (Table 12). The reported efficiency of phosphorus removal was 73.0–93.0% [54,55]. Further, Li et al. [56] analysed the impact of individual factors on limestone’s adsorption of P using a series of periodic experiments, showing increased removal efficiency with both temperature and contact time. However, where the initial concentration was higher, the process was less efficient. The size of the fraction had no significant impact, and the authors observed the maximum efficiency (above 90.0%) at a pH below 6.4, while pH values higher than 8.15 were associated with reduced efficiency [56].

Wollastonite is another reactive material in which the Ca/Mg sorption group predominates. Previous studies showed that when the initial concentration is lower, the sorption capacity is reduced drastically. This likely precludes the use of wollastonite in wastewater treatment [57] (Table 13). Removal efficiency is satisfactory when concentrations of P are higher, with 51.1% achieved in column experiments with 5 mg P/L [58]. Static experiments reported 90.0–93.0% efficiency with P concentrations of 14–61 mg/L [57].

The use of gravel in filtration systems likewise reflects the capacity to bind phosphorus, as Ca, Al, and Fe engage in adsorption and precipitation reactions. Sorption properties differ according to the content of these compounds (Table 14 offers a characterisation of these compounds according to the literature data). According to Vohla et al. [53], gravel has sorption capacities of 1.20–1.70 g P/kg. However, Mann et al. [59] only arrived at figures of 0.026–0.048 g P/kg—a reflection of the low content of Fe and Al ions in their material compared to the material tested by Vohla et al. [53]. Gravel of a lower fraction-size was also found to remove more phosphorus [53].

Reactive materials also include raw materials from the sea, such as oyster shells, clams, and crushed coral, which represent renewable sources of CaCO_3_. The materials researched by Zapater-Pereyra et al. [60] were used in raw form but also followed a pyrolysis process at 750 °C. Their experiments were performed on 500 mL solutions of phosphorus compounds, with 10 g of material, and the solutions were shaken at 100 rpm for 7 days. Materials previously subjected to pyrolysis were immediately capable of removing all phosphorus, with the efficiency of the process reaching 99.0%. The raw materials showed markedly lower efficiency—12.0–17.0%—after 12 days. Changes in pH following the mixing of the marine materials with a source of phosphorus were also investigated. The pH proved stable for raw materials (in the range of 7.1–7.8), while pyrolysis of the marine material indicated an increase in pH to 12.0. Studies further showed that an increase in pH to 12.0, achieved by adding NaOH to a solution, also immediately increases the efficiency of the phosphorus-removal process. Ultimately, the performance attained was similar to that with pyrolysed materials.

Research indicates that natural materials mainly remove P via an adsorption process, while heat-treated materials (with their relevant high-pH conditions) exert their main effects via precipitation. While the process for removing P may be highly efficient, the tested marine raw materials are likely to have limited involvement in wastewater treatment due to the need for the pH to be adjusted later [60,61]. Other research on the sorption capacity of powdered sun corals (with high concentrations of calcium carbonate) has studied raw sun corals (RSC), corals modified physically (SCA), and corals modified chemically (SCC) [62]. The maximum sorption capacities found for these three were 6.83, 7.06, and 9.60 g P/kg, respectively [62]. The group of marine materials able to bind phosphorus also includes “shell sand”, a naturally occurring carbonate material (which also includes some worn-down coral). As Table 15 shows, shell sand has been reported to have sorption capacities of 3.00–17.00 g P/kg, which was confirmed by Sovik et al. [63] with values of 0.80–8.00 g P/kg (against initial P concentrations of 5–1500 mg/L). The shell-sand analysis developed by Adam et al. [64] indicates that the lower the initial concentration is (across a range of 10–15 mg P/L), the lower the efficiency of P’s removal. Conversely, the higher the initial concentration is (across a range of 50–480 mg P/L), the higher the removal efficiency. The different sorption capacities obtained by Roseth et al. [65] likely resulted from the dissolution of calcium phosphate.

Column experiments have confirmed the efficiency achieved by shell sand to be around 3.50 g P/kg [65]. Studies using the powdered shells of freshwater clams by Xiong et al. [66] revealed that calcining the material at 700 °C for 20 min increases the material’s capacity to remove phosphate by 25.0% to 55.0% (at pH 5.5). For adsorption isotherm testing, the Temkin and D–R equations have been applied to all three shell types, indicating chemical adsorption. The maximum phosphorus adsorption capacity of natural clam shells is 6.95 g P/kg, while the removal efficiency for calcined and natural materials is 58.23% and 26.04%, respectively, at pH 5.5 [66].

## 3. Synthesized and Waste Materials and Their Modifications

Research into the use of waste materials agrees with the model of the circular economy, which assumes that manufactured products, as well as their raw materials, should remain in the economy for as long as possible, minimising the amount of waste generated [67]. Slag is waste from metallurgical processes involving metals, mainly steel and iron. Table 16 presents the test results for various types of slag according to their capacity to remove phosphorus. In a study by Xu et al. [68], the maximum sorption capacity determined for furnace slag, in line with the Langmuir model, was 8.89 g P/kg. Using column research, Dunets et al. [69] arrived at a similar value of 8.80 g P/kg for furnace slag (where the initial concentration of P was 60 mg/L). Other work on steel slag modified by high-powered ball-grinding from Li et al. [70] found adsorption capacities between 1.06 and 18.88 g P/kg, where phosphorus concentrations were 1–20 mg P/L. In contrast, according to Oguz [71], blast-furnace slag, as a by-product formed when iron ore is smelted, has 6.37 g/kg efficiency for binding phosphorus when the element is present at a concentration of 500 mg/L. The hydrated calcium silicate (CHS) obtained from blast-furnace slag has emerged as a very good phosphorus adsorbent. When initial concentrations are 38.0–47.5 mg P/L, this material can achieve a maximum adsorption capacity of 53.11 g P/kg. This adsorbent is particularly promising for wastewater and water since it exerts activity even when the phosphate concentration is low and has tolerance to a wide range of pH values [72]. However, Yasipourtehrani et al. [73] reported that samples of blast-furnace slag involved in the process release various concentrations of the toxic metals Al, Cd, Co, and Hg into solutions, a factor that could severely limit the material’s application in treatment.

Biochar, which is obtained through biomass pyrolysis, is characterised by a loose porous structure that encourages its use as a soil improver. It also has applications in wastewater treatment and environmental protection due to its adsorption and cation-exchange properties. Research by Qiu et al. [74] on modified biocarbon (pine wood subjected to pyrolysis at 500 °C in the presence of fly ash and gangue) found that modification increased both the specific surface area and the adsorbent capacity (Table 17). Analysis of the adsorption kinetics showed that the highest phosphorus binding rate was attained by biochar with gangue. The kinetics were described using pseudo-first-order and pseudo-second-order kinetic models, though the fit was better with the latter, indicating amounts of adsorbed phosphorus of 1.80–2.17 g P/kg. The maximum adsorption capacity according to the Langmuir model was 2.39 g P/kg for biochar only, 3.08 g P/kg when fly ash was also used, and 3.20 g P/kg with gangue [74].

Zhu et al. [75] obtained biochar coated with MgO nanoparticles by applying pyrolysis of MgCl_2_-impregnated corn straw. The adsorption process was best described using the Langmuir–Freundlich model, with a maximum sorption capacity of 60.95 g P/kg recorded. The kinetic analysis of biochar with MgO under different processing times indicated that phosphate adsorption on this material was mainly controlled by rapid binding with an external surface (about 75% of the adsorption equilibrium), while the rate was limited by the slow diffusion of phosphate into the biochar (about 25% of the adsorption equilibrium). The analysed dolomite-modified biochar was characterised by a maximum adsorption capacity of 29.18 g P/kg [76].

Lama et al. [77] experimented with coal slag, a by-product of thermal power plants. The particle size in this case was about 50 μm, while the SSA value was 9.20 m^2^/g. The maximum sorption capacity was 21.63 g P/kg, while the concentration range for P was 0–30 mg/L, and the other conditions included pH 6, an adsorbent dose equal to 0.1 g, and a contact time of 45 min.

The literature on static adsorption gives a maximum sorption capacity of fly ash equal to 0.86 g P/kg (with 20 g of material and solutions with concentrations between 2.5 and 40 mg P/L). The same authors, running a column experiment (with initial concentrations of 35–45 mg P/L and a process duration of 40 days), obtained a saturation of the material with phosphorus at a level of 0.30 g P/kg [14]. Research by Oguz [78] further showed that fly ash’s phosphorus sorption capacity (with a 0.12–0.002 mm fraction and 0.52 m^2^/g SSA) is greater with a higher temperature, mixing speed, pH, and phosphorus concentration in the solution. Fly ash has the ability to adsorb up to 71.87 g P/kg at an initial concentration of phosphorus equal to 130 mg P/L [78]. Research conducted by Xu et al. [68] gave a maximum sorption capacity for this material of 8.81 g P/kg (with 2 g of material + a solution with an initial concentration of 100–1000 mg P/L, shaken for 24 h at 200 rpm), which is in line with the Langmuir equation.

Autoclaved aerated concrete has been classified as a reactive material for phosphorus removal due to its high lime content. This material’s application is also favoured by its high content of tobermorite (a hydrated calcium silicate mineral). The sorption capacity was confirmed by tests showing the material’s capacity to reduce the amount of phosphorus by 70–80% [79]. Table 18 presents the sorption properties of this autoclaved aerated concrete [80,81,82,83,84]. The material has a satisfactory capacity for phosphorus sorption and can thus be used in the treatment of wastewater and water. However, an unfavorable feature is its alkaline pH.

Boujelben et al. [85] experimented on the sorption potential of three modified sorbents, synthetic iron oxide-coated sand (SCS), naturally iron oxide-coated sand (NCS), and iron oxide-coated crushed brick (CB). The material analyses (SEM Microscopy) showed that the crushed version differs from the others in having the most micropores and a larger specific surface area due to its clay content. The maximum phosphate sorption capacity at an optimal pH of 5.0 was 1.50 g P/kg for SCS, 1.80 g P/kg for CB, and 0.88 g P/kg for NCS. The effect of temperature on sorption was also investigated, with the results indicating that adsorption is an endothermic process [85].

Chen et al. [86] tested magnetic nanocomposites (magnetic diatomite (MDC) and aluminium lithium (MIC)) obtained by loading Fe_2_O_3_ nanoparticles onto the raw surfaces of materials using the solvothermal method. The highest adsorption capacity characterized an acidic solution. The adsorption kinetics suggested a pseudo-second-order equation, with limitations on the rate influenced by intramolecular diffusion. The maximum values noted for phosphorus adsorption capacity in MDC and MIC were 11.89 and 5.48 g P/kg, respectively. The mechanisms underpinning adsorption are the electrostatic attraction between the adsorbent and adsorbate and ligand exchange. MDC and MIC show appropriate selectivity and regenerative capacity for reuse [86].

Ma et al. [87] used inorganic–organic montmorillonites (IOMts) obtained by modifying polyhydroxy-aluminium (Al_13_)-pillared montmorillonite (AlPMt) with a cationic surfactant (C16) and a zwitterionic surfactant (Z16). Analysis of the phosphorus adsorption process using these materials showed that, for concentrations in the 20–140 mg P/L range and a solution with a pH of 5.0, the sorption capacity ranged between 10.07 and 13.04 g P/kg, depending on the type of modification [87]. 

Goethite is another reactive material, a version of which was synthesised under laboratory conditions and analysed in studies conducted by Siwek et al. [9]. The adsorption obtained was in the range of 6.10–8.00 g P/kg and better at 10–20 °C than at 4 °C (10 mg of goethite + 25 mL of a solution at an initial concentration of 10 mg P/L). The process of adsorption described by the Langmuir model entails a maximum sorption capacity for goethite in the range of 18.20–27.00 g P/kg, depending on the type of solution. A higher capacity was observed when a solution including natural water from bodies of water affected by eutrophication was used instead of distilled water. This points to the presence of other ions capable of affecting the process by which phosphate ions are inactivated [9]. Goethite is apparently able to bind P effectively over a wide pH range (from 4.0 to 11.0) [9,88].

Maamoun et al. [89] conducted research on permeable reactive barriers (PRBs) with a nano-zero-valent iron (nZVI), which is another putative synthesized material for phosphorus removal from wastewater. Static experiments were conducted at pH = 7 ± 0.5 under anaerobic conditions. Solutions with initial concentrations of 5–1000 mg P/L including 1 g/L of nZVI were shaken at 300 rpm. The maximum adsorption capacity of nano-ferrous particles for phosphorus was 54.34 g P/kg-Fe, while that for the mixture (1%) of Fe0 + river sand was 3.9 g P/kg. In addition, the calculated correlation coefficients indicated that the Langmuir isotherm is more appropriate for describing the adsorption process than the Freundlich model. Column experiments were carried out via the in situ method for one month using three columns of sandy soil with different configurations of layers that reacted with the nZVI barriers (column C1: 1 large reactivated layer, column C2: two small reactivated layers, and column C3: river sand without a reactivated layer). Solutions with an initial concentration of 25 mg P/L were introduced into the columns, and the leachate was collected for analysis. It was found that two layers of the reactive mixture (Fe^0^/river sand) offered comparatively better phosphorus removal performance than one combined layer. The highest overall phosphorus removal efficiency (68%) was achieved for the C2 column over 29 days of experimentation. The authors noted that the phosphorus removal mechanism of nZVI nano-valent iron barriers could rely on both the chemical adsorption of phosphorus on the iron oxide layer (resulting in hydroxide as a result of corrosion of the core nZVI) and the co-precipitation of iron ions on the surfaces of particles. Due to its high sorption capacity, this material can be successfully used for in situ remediation techniques and also in other technologies for phosphorus removal from wastewater [89,90]. Table 19 presents a list of the sorption properties of the synthesized materials.

Portland cement is a type of hydraulic mineral binder obtained by mixing a ground cement clinker with gypsum (acting as a setting time regulator) in an amount up to 5%. Cement clinker is produced by burning (raw) materials containing limestone and aluminosilicates. The basic components of Portland cement are mixed in a cement mill, and the material can be clean or multi-component (supplemented with slag or limestone). After determining the respective phosphate binding capacities of these two types, Masłoń [91] found both forms promising for use in wastewater technology. Trials with a synthetic solution (initial concentration 1.2 mg P/L) and wastewater (initial concentration 1.7 mg P/L) provided removal efficiency values of 97.2 and 96.3%. On the other hand, testing of the pure cement revealed 95.0% efficiency with wastewater (initial concentration 1.2 mg P/L) and a corresponding value of 96.8% for the synthetic solution. Given that both forms of Portland cement proved advantageous, phosphate removal was deemed to be based mainly on the lime-precipitation principle. However, the usefulness of Portland cement in removing phosphorus from wastewater has its limitations, as it is necessary to correct the pH due to the material’s strong alkalizing properties [91]. Other tests carried out on pure Portland cement revealed a maximum sorption capacity of 83.00 g P/kg, as determined using the Frumkin isotherm model [92].

Brick dust is obtained by mixing ground brick waste with ground red clay at a ratio of 1:10. For this material, Masłoń et al. [93] found a maximum sorption capacity (determined according to the Langmuir model) of 0.455 g P/kg. Table 20 shows the properties of brick dust compared to powdered expanded clay. Brick dust achieved 54.7 ± 8.8% removal of phosphorus compounds from wastewater (initial concentration 3.1 mg P/L, maximum adsorbent dose equal to 5 g/L) [93].

The specific industrial wastes presented in Table 21 have also been studied from the perspective of phosphorus compounds in wastewater. For example, wastes from the treatment of acid mine waters are used in neutralising acidity and removing dissolved Fe. Penn et al. [94] considered two acid mine-drainage treatment residuals with different chemical compositions (ADMR1 and ADMR2), obtaining respective maximum sorption capacities of 40.00 and 29.00 g P/kg. This difference reflected the more favourable Ca content in ADMR1.

Another industrial waste is water treatment sludge (WTR), which contains large amounts of Al due to the use of aluminium sulphate as a coagulating agent. The results for these sludges suggest an adsorption capacity around 32.00 g P/kg. The composition in this case is dominated by the Fe/Al sorption group, indicating a process based mainly around adsorption [94].

Gypsum is a product of flue-gas desulphurisation, which mainly includes calcium and is thus a mechanism based primarily on the precipitation of calcium phosphates. The phosphorus adsorption capacity in this case was 14.00 g P/kg, with a low pH (of 4.5) found to significantly reduce efficiency [94].

Red mud is a by-product that was formed during bauxite leaching in the Bayer trial. This waste raises serious storage-related environmental problems due to its large quantities and alkaline nature. The P-removal capacities were tested for raw red mud versus raw fly ash, as well as their acid-activated and heat-treated forms. Table 21 and Table 22 summarise the relevant results. Li et al. [95] shook 20 mL of KH_2_PO_4_ solution (initial concentration 155 mg P/L) at 180 rpm with 0.1 g sorbent for 4 h at 25 °C. In parallel, they also studied the effects of the acidification or calcination of the raw materials on phosphate sorption. The results indicated that the materials calcined at 700 °C offered the greatest efficiency. With acid activation, the optimal version involved 0.25 mol/L HCl. Activated red mud in the two variants achieved a P-removal efficiency of 99.0%, while the raw-form’s efficiency was 47.8%. Fly ash achieved a 45.2% removal efficiency following acid activation and 52.9% after undergoing heat treatment. The level for the raw form of fly ash was 16.1%. The Freundlich and Langmuir models were used to simulate the sorption equilibrium, with a better correlation in the latter context. Red sludge activated by heat treatment at 700 °C proved the most efficient at removing phosphorus, presenting the highest sorption capacity [95]. Using the same material, Penn et al. [94] found sorption capacities of 25.00 and 29.00 g P/kg for red mud and fly ash, respectively (see Table 22).

Research has also sought to determine the capacity to remove phosphorus by ash from the clinker, as well as in the iron oxide tailings from copper and nickel mines [96]. The results are shown in Table 23. Iron oxide tailings were found to have a greater capacity to remove P (at 1.29 g P/kg) compared to the clinker ash (0.29 g P/kg). However, the latter has more suitable physical properties for the adsorption process. Testing involved 100 mL of domestic sewage, with various doses of adsorbent added prior to 24 h of shaking on an orbital shaker at 120 rpm at room temperature. Data were set against linear forms of the Langmuir and Freundlich equations, with the former offering the best fit for the determination of sorption capacity. Analysis also showed that iron oxide tailings have a higher regenerative potential than clinker ash, suggesting that they are the better adsorbent [96].

Some wastes from the agri-food industry show phosphorus sorption properties. For example, *Arachis hypogaea* husks contain polysaccharides. Unmodified husks have a negative charge in the solution but possess a positive charge after ammonization [97]. The sorption capacity was determined by adding sorbents at a dose of 5 g/L to 200 mL of solutions with concentrations from 0.1 to 5 mg P/L, which were shaken for 1 h. The Freundlich, Langmuir, and double Langmuir models were used to describe the results. The Langmuir model showed the best fit, based on which the maximum sorption capacity was determined: 0.01 g P/kg for unmodified husks and 0.10 g P/kg for modified husks. Therefore, the amino groups introduced during modification are the main factor that causes phosphorus sorption. Table 24 shows the optimal pH values of phosphorus removal for husks and solution reactions that can be obtained after this process. The neutralization ability of husks is crucial when they are used in wastewater treatment plants [97]. Studies on the sorption capacity of phosphorus by chitin and chitosan flakes have also confirmed that the adsorption capacity depends on the amount of amino groups [98]. According to Table 24, the maximum sorption capacity determined according to the Langmuir model was 6.64 g P/kg for chitosan and 2.09 g P/kg for chitin. The better efficiency of chitosan in removing phosphorus is because chitosan has more amino groups on its surface than chitin does. In addition, the optimal pH for phosphorus removal by chitin is 3.0, with a final solution pH of 3.3. For chitosan, the optimum pH for the process is 4.0. However, due to the ability to neutralize the solution, a final pH of 6.75 can be obtained; thus, pH correction is not required after the adsorption process [98].

## 4. Man–Made Materials

Man-made materials are materials with precisely fixed chemical compositions that have been commercialised and are sold in bulk.

*Polonite*^®^ is sedimentary opoka heat-treated at 900 °C to convert CaCO_3_ into the more-reactive CaO. *Polonite*^®^ usually has a 2.0–6.0 mm fraction. Kaczmarczyk et al. [99] provide an estimated phosphorus adsorption capacity of 40.90 g P/kg. They also reported on column experiments, finding phosphorus-removal efficiencies of 52.8% for wastewater (initial concentration 10.69 mg P/L) and 72.4% for synthetic solutions (initial concentration 4.41 mg P/L). Other, long-term experiments confirmed phosphorus removal efficiency by *Polonite*^®^ to be around 97.0% for synthetic solutions and 92.0% for wastewater. This indicates that the other impurities present in wastewater have an impact on the adsorption process [100].

*Leca*^®^ is a lightweight aggregate obtained from expanded clay. Kaczmarczyk et al. [99] obtained an estimate for phosphorus adsorption capacity equal to 5.10 g P/kg. The removal efficiency noted in their column experiments was 31.7% for wastewater (initial concentration 10.69 mg P/L) and 28.3% for synthetic solutions (initial concentration 4.41 mg P/L) [99]. The powdered expanded clay fraction was tested by Masłoń et al. [93,101], with the result providing a maximum sorption capacity of 0.593 g P/kg (Table 20). When the product was used on wastewater (initial concentration 3.1 mg P/L), a phosphorus removal efficiency of 80.7 ± 8.7% was obtained with a maximum adsorbent dose of 5 g/L. A slight increase in pH from 7.56 to 7.69 was also noted. Expanded clay also has the ability to improve the sedimentation of activated sludge and biogranulation in a granular sequencing batch reactor [102,103,104].

Lightweight aggregates (LWAs) can be clay or shale [105]; the production process involves clay aggregates passing through a rotary kiln at 1200 °C. Tests on this material differ significantly from each other in terms of their results, as both origin and chemical composition significantly affect the amount of sorption. The highest level of sorption observed (12.00 g P/kg) was given by Jenssen et al. [106]. Compared to the analysis carried out by Zhu et al. [105], where the sorption capacity was 3.47 g P/kg, the better efficiency of Jenssen et al. likely resulted from a higher content of dolomite [106].

*Rockfos*^®^ is a waste material obtained when opoka is heat-treated above 700 °C. Kasprzyk et al. [107] gave the high maximum phosphorus sorption capacity for this material (equal to 256.40 g P/kg).

*Pollytag*^®^ is an aggregate made from fly ash via granulation and sintering at a temperature of 1000–1350 °C. An analysis by Bus et al. [108] showed that, at higher initial concentrations, *Pollytag*^®^ is characterised by a higher efficiency of phosphorus removal. At concentrations of 1–3 mg P/L, the efficiency of phosphorus removal is 1.0–2.5%. However, at an initial concentration of 10 mg P/L, the efficiency achieved is 34.0%. *Pollytag*^®^ is thus a good reactive material for removing phosphorus from wastewater in cases where concentrations of phosphorus are in the 8–19 mg P/L range. The sorption capacity of this material, determined according to the Langmuir model, was 32.24 g P/kg [108].

*Filtralite*^®^ is a new-generation LWA made in Norway from natural ilitic mineral clay with natural additives. Adam et al. [64] showed how, at an initial concentration of 480 mg P/L, the adsorption capacity is only 2.50 g P/kg. However, compared with other materials, *Filtralite*^®^ was shown to be a more effective remover of phosphorus when initial concentrations were lower, which is an important finding from the perspective of wastewater treatment. Column experiments showed a >90.0% sorption capacity for the tested material in wastewater compared to 54.0% for the synthetic solutions (perhaps due to the presence of other ions and the formation of a biofilm) [64].

*Filtra P* is produced by heating materials such as limestone, gypsum, and iron oxides. Analysis of this material by Jourak et al. [109] showed that, where initial concentrations were in the range of 3–100 mg/L, phosphorus in the solution was removed completely and bound quickly to the material. The maximum adsorption capacity was 4.30 g P/kg (with an initial concentration of 300 mg P/L).

Table 25 compares the properties of the best known examples of man-made materials for the removal of phosphorus from wastewater by sorption and precipitation.

## 5. Summary

The present analysis of sorption properties included natural, waste, synthesized, and commercial materials, as well as modified versions thereof. A careful review of previous research can determine the impact that the chemical and physical compositions of these materials, as well as their process conditions, have on the ability to bind phosphorus. As the experimental conditions applied were variable, each material was analysed separately to determine each factor’s potential effects on sorption capacity.

The literature shows that the search for effective reactive materials is always focused on natural materials. Sorption tests carried out on these materials determine whether industrial waste containing a given natural substrate could, in the future, be used to remove P from wastewater and whether synthetic modifications of the materials make sense. Analysis of the properties of the natural materials also determines the effective substrates for the modification processes by which the sorption properties of other materials could be improved.

Natural materials are characterised by their lower removal efficiency (sorption capacity) of phosphorus compounds from various solutions (52.02 g P/kg for dolomite, 10.70 g P/kg for diatomite, 26.04 g P/kg for sand from shells, and 19.60 g P/kg for opoka) than their modifications using thermal or chemical treatments. Thus, for example, the sorption capacity of roasted opoka reaches a level of 181.82 g P/kg. However, raw waste materials, depending on their location, prove highly efficient at removing P (with red mud at 345.50 g P/kg, wastes from the treatment of acid mine water ar 40.00 g P/kg, fly ash at 78.44 g P/kg, blast-furnace slag at 75.70 g P/kg, and autoclaved aerated concrete at 12.25 g P/kg). Synthesized materials, due to their ability to possess the necessary properties, are characterized by a high sorption capacity (e.g., nano-valent iron barriers (nZVI), 54.34 g P/kg).

Other materials modify natural and raw waste materials in combination, with an example being biochar + dolomite, which can achieve 29.18 g P/kg sorption. Materials can also be improved by applying nanoparticles of appropriate compounds to their surfaces. Biochar coated with MgO nanoparticles achieved a sorption-capacity value of 60.95 g P/kg. Commercialised man-made materials obviously achieve a high efficiency of phosphorus removal, as does *Polonite*^®^, with a 40.90 g P/kg sorption capacity and *Rockfos*^®^, with a capacity of 256.40 g P/kg.

Since the adsorption process is not yet fully understood, and there are many methods for testing potential sorbents, it remains very difficult to engage in meaningful comparisons between different materials. Furthermore, adsorption is a process that is sensitive to any change in conditions. Any analysis needs to account for the circumstances in which a process occurs and for the impact of many different factors. When a reactive material is chosen, the pattern followed must provide a rational determination of each given substrate’s properties. The physical parameters and chemical composition should be determined first, before the adsorbent dose and adsorbate concentration are selected in line with the potential application. The next step involves static tests that are conducted to determine the sorption capacity and kinetics of the adsorption process, followed by column and field tests performed to confirm the static tests [84].

The analysis provided here shows the higher sorption capacities of materials whose initial phosphorus concentrations are greater. The exception to this rule is shale, which showed an opposite trend [23,24]. The results in the literature further suggest a higher process efficiency with a higher dose of the adsorbent.

However, the selection of reactive materials for use in the treatment of wastewater necessitates consideration of the P-removal efficiency, as the concentrations of P should correspond to those present in real wastewater. The pH for the process must also be adequate and adjusted as necessary during the process line of a given wastewater treatment plant.

The economic aspects of using a material in the technological process of a sewage treatment plant must also be considered. The present analysis shows that modified materials offer the best efficiency. Notably, all modifications or improvements (through calcination, particle application, or the addition of other reagents) entail costs that affect the final price of the product. When planning the modernization or construction of a wastewater treatment plant, the market value of the material should be estimated, and the possibility of its future changes should be assessed. This is done to determine the cost-effectiveness of using the technology with a given material. In addition to modification costs, the price of the material is also influenced by the size of the available resources and the cost of transport.

Due to the wide variety of research methods used, as well as the different chemical compositions, it is difficult to determine a perfect comparison of materials based on their efficiency in removing phosphorus from wastewater. Such confirmation may also come from differences in the results of tests using the same natural materials, whose chemical compositions are slightly different due to the places of their extraction. Therefore, each material should be approached individually, with a proper selection of research conditions in line with the potential application.

### 5.1. The Influence of a Material’s Chemical Composition, pH and Physical Properties on Phosphorus Sorption

For removing phosphorus from wastewater or other water, any potential reactive material should have a chemical composition rich in compounds that can retain P—i.e., Ca, Mg, Fe, and Al. Considering the method by which these compounds bind phosphorus, a distinction must be drawn between materials with dominant sorption groups (either Al/Fe or Ca/Mg). The properties differ here, with Ca playing the key role within the Ca/Mg sorption group, which precipitates P in the form of sparingly soluble compounds. The research cited here shows that materials with Ca in the form of CaCO_3_ have a lower phosphorus sorption capacity than substrates with CaO or Ca(OH)_2_ [47]. However, thermal treatment raises the sorption capacity, as a high-temperature conversion of calcium carbonate to calcium oxide and carbon dioxide occurs. This is true of opoka, whose sorption capacity after calcination increases by ca. sixfold [41]. A similar factor applies to marine materials like oyster shells, mussels, and corals, whose effectiveness in removing phosphorus increases from six- to eightfold via pyrolysis [60]. Heat-treated marble likewise possesses altered Ca content (risen from 32.9% to 48.2%), with phosphorus binding shown to increase from 103.20 to 181.20 g P/kg [37]. For red mud with CaO equal to 46.0%, the sorption capacity increased threefold following calcination (to 345.50 g P/kg). This was, in fact, the highest value noted for any of the materials presented [95].

Notably, the use of materials with high Ca content in phosphorus removal increases the solution pH from 8.0 to 13.0. Where practical wastewater treatment is involved, this factor will necessitate pH correction via additional processes like dilution or aeration. Another problem with filters using reactive materials containing dominant Ca content involves plugging via cement formation as CaCO_3_ crystallises. This problem can be solved by removing CO_2_ or by using carriers over 5 mm in size [48].

The Al/Fe sorption group participates in a phosphorus-binding process in which there is an exchange of the ligands of hydrolysed complexes (formed due to the presence of aluminium or iron ions). The sorption capacity of materials containing this group increases over the pH range from neutral to acidic [13], which was confirmed, for example, for aluminium hydroxide (whose maximum adsorption occurs at pH values between 4 and 6). Vohla et al. [48] state that any process involving sands or gravels at pH values above 6 combines the adsorption of iron and aluminium on oxides, while the process at lower pH values entails the precipitation of iron and gallium phosphates (strengite, variscite). Among the presented materials containing dominant Al/Fe groups, products based on fly ash offer the greatest sorption capacity. *Pollytag*^®^ provided a phosphorus binding capacity of 34.00 g P/kg, while fly ash activated with 0.25 M HCl offered a value of 78.44 g P/kg.

While the most important physical properties of materials are porosity and specific surface area, the material’s use as a filtration medium determines all the parameters that can affect functioning. The assumption that the greater SSA is, the better the contact between the adsorbent and the pollutant will be was confirmed for most of the materials presented. Only for akadama clay did a raw material with a greater SSA (equal to 117.67 m^2^/g) present a lower sorption capacity (5.88 g P/kg) than a material modified with SSA (down to 75.27 m^2^/g, for a sorption capacity of 9.19 g P/kg).

Acid modification was shown to increase the pore volume and thus the active sites, thereby increasing the sorption capacity [19]. This confirms that each material must be considered individually.

### 5.2. Possible Use of Reactive Materials in Fertiliser Production

Reactive materials with adsorbed phosphorus can be reused in agricultural production—for example, to increase the capacity of soil to retain water or to ensure a supply of plant nutrients. Equally, phosphorus compounds removed from wastewater through selective sorption in the solid phase can be directly used as fertilisers or soil conditioners. A second option involves recovering P from reactive materials via chemical precipitation with salts of Mg or Ca, thereby producing a high-purity fertiliser [111]. P-enriched materials can also be used in agriculture when the form is plant-available. The presence of toxic compounds and pathogens limits any potential use in agriculture—e.g., due to high levels of leaching metals and metalloids. In addition, materials should have high permeability to avoid clogging. Ca-related forms of phosphorus are more plant-available than their Al or Fe-related forms.

Generally, however, any broader view taken as new reactive materials are sought should entertain the possibility of ultimate reuse in agriculture, which complies with the assumptions of the circular economy.

## 6. Conclusions

This paper has reviewed the materials able to bind phosphorus via sorption and/or precipitation.

Due to the variety of materials and their properties, it is not possible to draw uniform conclusions for all tested materials. Among the natural materials, calcined opoka achieved the highest sorption capacity of 181.20 g P/kg. Among the waste materials, red calcined sludge proved the most efficient at sorbing phosphorus, with 345.02 g P/kg. Within the group of commercial materials, *Rockfos*^®^ demonstrated the highest sorption capacity (with a reported value of 256.40 g P/kg). Among the synthesized materials, nano-valent iron (nZVI) barriers offered the best sorption capacity at 54.34 g P/kg. Natural materials have been the most commonly studied, as these materials represent a starting point for the waste materials to be tested, as well as the modifications of materials. Indications for further research in the field are also supplied in this paper. As reactive materials are sought, the matter of sorption capacity vis-à-vis phosphorus should be supplemented by a consideration of physical properties more generally (and the potential for contamination). Cost and availability are also of obvious importance given the need for economically viable materials.

Considering the ongoing demand for phosphorus compounds and the lack of synthetic substitutes, new materials should be sought in the perspective of using their form with adsorbed phosphorus in the production of fertilizers. In this case, materials with a predominant Ca/Mg sorption group are more useful because of their easier accessibility for plants. Further research should also assess materials for their sorption of heavy metals and for the general presence of toxic compounds and pathogens likely to limit any use in agriculture.

## Figures and Tables

**Table 1:** Sorption properties of various forms of bauxite.

Type of Phosphate	Bauxite ^(*)^ Mixed with Distilled Water and Dried [15] ^(**)^		Raw Bauxite ^(*)^ [16] ^(**)^			Activated Bauxite at 600 °C [16] ^(**)^		
	pH [–]	Phosphorus Removal Efficiency [%]	pH [–]	Phosphorus Removal Efficiency [%]	Sorption Capacity [g P/kg]	pH [–]	Phosphorus Removal Efficiency [%]	Sorption Capacity [g P/kg]
Orthophosphate	4.4	67.30	4.50	67.3	0.67	4.5	97.9	0.98
Glycerophosphate	5.4	57.71	3.20	39.9	0.40	3.2	90.9	0.91
Tripolyphosphate	3.2	39.98	5.40	57.7	0.58	5.4	97.5	0.97

^(*)^ Material composition: Fe_2_O_3_—16.95%; Al_2_O_3_—56.91%; SiO_2_—8.72%; CaO—0.91%, ^(**)^ Description of the study: 1 g material + 100 mL solution with an initial concentration of 10 mg P/L; contact time: 2 h; speed: 400 rpm; fraction: <0.074 mm.

**Table 2:** Studies on the sorption properties of diatomite.

Diatomite [17] ^(*)^		FerrihydriteModified Diatomite [17] ^(**)^		LanthanumModified Diatomite [18] ^(***)^
pH [–]	Sorption Capacity According to the Langmuir Model [g P/kg]	pH [–]	Sorption Capacity According to the Langmuir Model [g P/kg]	Phosphorus Removal Efficiency [%]
4.0	10.20	4.0	37.30	98.2
8.5	1.70	8.5	13.60	

^(*)^ Material composition: Fe_2_O_3_—1.50%; Al_2_O_3_—4.00%; MgO—0.30%; CaO—0.50%, ^(**)^ Material modification with ferrihydrite at a quantity of 0.24 g Fe/g. Description of the study: 100 mL solutions with initial concentrations of 0–40 mg P/L + 0.05 g material, shaken at 200 rpm for 72 h, ^(***)^ Description of the study: 0.08 g material + solution with an initial concentration of 5 mg P/L.

**Table 3:** Comparison of the properties of natural and modified akadama clay.

References	Material		Specific Surface Area [m^2^/g]	Sorption Capacity According to the Kinetic Model [g P/kg]	Maximum Sorption Capacity According to the Langmuir Model [g P/kg]
[19]	akadama clay	activated H_2_SO_4_	75.27	3.95	9.19
		natural	117.67	3.41	5.88

**Table 4:** Sorption properties of laterite in natural or modified versions.

Laterite [22] ^(*)^		Acidified Laterite, ByProduct of the Production of Ferric Aluminium Sulphate [20] ^(**)^		Laterite [21] ^(***)^	
Initial Phosphorus Concentration [mg P/L]	Phosphorus Removal Efficiency [%]	Initial Phosphorus Concentration [mg P/L]	Sorption Capacity [g P/kg]	Temperature [°C]	Sorption Capacity [g P/kg]
50	60.0	0.035	1.65	24.85	1.07 ^(L)^
5–10	80.0–90.0	5–50	2.73 ^(L)^	34.85	1.14 ^(L)^

^(*)^ Material composition: Fe_2_O_3_—26.70%; Al_2_O_3_—39.80%; description of the study: 10 g material (fraction 2.0–3.4 mm) + 100 mL solution, shaken at 65 rpm, ^(**)^ Description of the study: material 1 g/L + 50 mL solution, shaken for 24 h at 100 rpm, ^(***)^ Material composition: Fe_2_O_3_—14.20%; Al_2_O_3_—24.40%; MgO—0.73%; CaO—0.74%; description of the study: 50 mL solutions with initial concentrations of 5–30 mg P/L + 0.3 g material, shaken for 12 h at 170 rpm, ^(L)^ Sorption capacity according to the Langmuir model.

**Table 5:** Sorption properties of shales.

References	Material	Porosity [%]	Type of Experiment	Hydraulic Conductivity [m/s]	Fraction [mm]	Initial Phosphorus Concentration [mg P/L]	Sorption Capacity [g P/kg]
[14]	Shale	38	column	0.001	6.8–12.6 ^(**)^	45	0.40
				0.001	6.8–12.6 ^(**)^	100	0.75
			static ^(*)^	0.001	6.8–12.6 ^(**)^	2.5–40	0.65 ^(L)^
[23,24]	Shale	70	static ^(***)^	0.0009	2.0	0–1000	0.17 ± 0.01
				0.0076	2.0–4.7	0–1000	0.14 ± 0.01
			static ^(****)^	0.0009	2.0	0–100	0.50 ± 0.04
				0.0076	2.0–4.7	0–100	0.25 ± 0.04
[25]	Shale	50	static ^(*****)^	0.032	< 0.4	5–250	0.51 ^(L)^
						5	0.06
						25	0.023
			column	0.032	1.0–2.0	25	0.17

^(*)^ Description of the study: solution + 20 g material, shaken at 60 rpm for 24 h, ^(**)^ Fraction 6.8–12.6 mm: 41.9% of all material, ^(***)^ Description of the study: solution + 3 g material, shaken for 24 h, centrifuged at 5000 rpm, ^(****)^ Description of the study: solution + 1 g material, shaken for 24 h, centrifuged at 5000 rpm, ^(*****)^ Description of the study: solution + dose of material 0.08 g/L, shaken at 300 rpm for 24 h, ^(L)^ Sorption capacity according to the Langmuir model.

**Table 6:** Sorption properties of zeolite, or modified versions of it.

References	Description of the Study	Material	Maximum Sorption Capacity According to the Langmuir Model [g P/kg]
[14]	solutions with initial concentrations of 2.5–40 mg P/L+ 20 g material (fraction: 6.8–12.6 mm), shaken at 60 rpm for 24 h	zeolite	0.46
[27]	solutions with initial concentrations of 500–10,000 mg P/l + 3 g material, shaken for 48 h at 100 rpm, centrifuged at 5000 rpm for 10 min	zeolite	2.15
[13]	solutions with initial concentrations of 0.5–1000 mg P/L + 0.4 g material, shaken for 24 h	zeolite synthesized from fly ash ZFA ^(*)^	35.31
		zeolite ^(**)^	2.19
	0.4 g material + solution with an initial concentration of 1000 mg P/L, shaken for 24 h	ZFA–Ca	54.17
		ZFA–Fe	31.75
		ZFA–Al	30.46
		ZFA–Mg	32.79

^(*)^ Material composition: Fe_2_O_3_—9.00%; Al_2_O_3_—18.90%; SiO_2_—34.40%; MgO—1.00%; CaO—7.30%, ^(**)^ Material composition: Fe_2_O_3_—0.20%; Al_2_O_3_—11.00%; SiO_2_ —69.50%; MgO—0.10%; CaO—2.60%.

**Table 7:** Comparison of sorption properties among variously modified types of bentonite.

References	Material	Specific Surface Area [m^2^/g]	Maximum Sorption Capacity According to the Langmuir Model [g P/kg]
[29]	hydroxyAl–modified bentonite	200.00	12.70
	hydroxyFe–modified bentonite	143.00	11.20
	hydroxyAl-Fe–modified bentonite	94.90	10.50
[28]	lanthanum(III)–modified bentonite	115.00	14.00

**Table 8:** Sorption properties of dolomite.

References	Description of the Study	Type of Medium	Initial Phosphorus Concentration [mg P/L]	Sorption Capacity [g P/kg]
[34]	column: material < 0.074 mm, flow rate 1 mL/min	distilled water	0.28	0.06
		ground water	0.34	0.07
		tap water	0.34	0.05
	static: solution + 5–40 g material, shaken at 200 rpm	synthetic solution	9.60	0.93
[33]	static: 0.2 g material + 100 mL solution, shaken at 90 rpm, centrifuged at 3750 rpm	synthetic solution	10.00–60.00	9.74–52.91 ^(*)^
			10.00–60.00	7.34–51.02 ^(**)^

^(*)^ Temperature of conducting the experiment: 20 °C, ^(**)^ Temperature of conducting the experiment: 40 °C.

**Table 9:** The sorption capacities of marble under different process conditions.

References	Material	Initial Phosphorus Concentration [mg P/L]	Diameter d_50_ [µm]	Composition CaO [%] MgO [%]		Final pH [–]	Sorption Capacity [g P/kg]
[37]	marble ^(*)^	1000	32.1	46.10	1.24	9.45	103.20
	calcined marble ^(**)^	1000	28.9	67.45	2.35	12.27	181.20
[38]	marble ^(***)^	100	22.6	46.06	1.24	–	17.00

^(*)^ Description of the study: 1 g material + 100 mL solution, shaken at 400 rpm for 16 h, pH = 5.0, ^(**)^ Description of the study: 1 g material + 100 mL solution, shaken at 400 rpm for 3 h, pH = 5.0, ^(***)^ Description of the study: reactor CSTR: dose of material 12 g/L, contact time 8.8 h.

**Table 10:** Comparison of the sorption capacity of opoka, depending on the method of calcination.

References	Material	pH [–]	The Ratio of Components SiO_2_/CaO	Fraction [mm]	Sorption Capacity [g P/kg]
			[%]/[%]		
[47]	Opoka	8.3	34.00/28.00	0.25–2.00	0.10
[41]	Opoka	6.8	44.65/23.75	0.25	19.60
[42]	Calcinated opoka at 900 °C	–	57.24/23.86	0.05–20	12.30
[41]	Calcinated opoka at 1000 °C	12.0	39.36/42.07	0.25	119.60
[43]	Calcinated opoka at 900 °C	12.4	66.57/30.91	dust	79.37
[43]	Calcinated opoka at 900 °C	12.4	45.00/58.76	dust	181.82

**Table 11:** Properties of sands in removing phosphorus from wastewater.

References	Diameter Material Grains		Composition CaO [%] MgO [%] Fe_2_O_3_ [%] Al_2_O_3_ [%]				Sorption Capacity at Initial Concentration 320 mg P/L [g P/kg]	Maximum Sorption Capacity According to the Langmuir Model ^(*)^ [g P/kg]	Phosphorus Removal Efficiency in a Column Experiment ^(**)^ [g P/kg]
	d_60_ [mm]	d_10_ [mm]							
[51]	3.20	0.80	9.79	0.35	0.68	0.48	2.68	0.061	0.134
	0.60	0.21	4.90	0.17	0.52	0.45	1.68	0.130	0.117
	3.40	0.61	8.72	0.21	0.51	0.36	3.94	0.064	0.165

^(*)^ Description of the study: 100 mL solutions with initial concentrations of 2.5–300 mg P/L + 5 g material, shaken for 20 h, ^(**)^ Description of the study: solution with initial concentration of 10 mg P/L, flow 240 mL/d, time 20 h/d for 12 weeks.

**Table 12:** Analysis of sorption by limestone.

References	Description of the Study:	Fraction [mm]	pH [–]	Maximum Sorption Capacity According to the Langmuir Model [g P/kg]
[14]	solutions with initial concentrations of 2.5–40 mg P/L + 20 g material, shaken at 60 rpm for 24 h	12.7	7.8	0.68
[47]	solutions with initial concentrations of 5–25 mg P/L + 1 g material	0.3–2.0	8.9	0.25
[54]	solutions with initial concentrations of 5–150 mg P/L + 1 g material, shaken at 160 rpm for 24 h	d_60_: 7.0 d_10_: 3.5	–	1.09

**Table 13:** Properties of wollastonite for phosphorus removal.

References	Composition CaO [%] MgO [%] Fe_2_O_3_ [%] Al_2_O_3_ [%]				Initial Phosphorus Concentration [mg P/L]	Sorption Capacity [g P/kg]	Phosphorus Removal Efficiency [%]
[57]	46.00	0.47	0.07	–	0.8–1700	0.0001–12.000 ^(*)^	–
					14–61	–	90.0–93.0 ^(*)^
[58]	21.14	2.21	3.07	10.31	5	–	51.1 ^(**)^

^(*)^ Description of the study: 75 mL solutions with initial concentrations of 0.8–1700 mg P/L + 10 g material, shaken at 200 rpm for 0.5–20 h, ^(**)^ Description of the study: column experiment: solution with an initial concentration of 5 mg P/L; flow 610 L/m^2^ for 68 weeks.

**Table 14:** Analysis of gravel sorption.

References	Composition CaO [%] MgO [%] Fe_2_O_3_ [%] Al_2_O_3_ [%]				Fraction [mm]	Sorption Capacity [g P/kg]	Phosphorus Removal Efficiency [%]
[59] ^(*)^	–	0.08	0.78	0.40	5.0–10.0	0.0258	–
	–	0.01	0.39	4.50	3.0–5.0	0.0478	–
[53] ^(**)^	19.60	6.80	1.12	0.66	0.9–1.8	1.20	32.6
	2.80	1.99	1.37	0.94	0.07–1.0	1.70	50.3

^(*)^ Description of the study: 40 mL solutions with initial concentrations of 5–100 mg P/L + 20 g material, shaken at 1500 rpm for 24, 30 h, ^(**)^ Description of the study: 75 mL solutions with initial concentrations of 5–100 mg P/L + 3 g material, shaken for 24 h.

**Table 15:** Research on the adsorption of phosphorus by shell sand.

References	Material	Composition CaO [%] MgO [%] Fe_2_O_3_ [%] Al_2_O_3_ [%]				Sorption Capacity [g P/kg]	Phosphorus Removal Efficiency in a Column Experiment [%]
[65]	powdered shell sand	39.00–42.00	0.60–3.20	–	–	14.00–17.00 ^(*)^	–
						3.00–4.00 ^(**)^	
[66]	natural powdered shells	52.60	0.20	0.15	0.14	6.95	26.0
	pyrolyzed powdered shells	55.20	0.21	0.03	0.08	–	58.2
[64]	shell sand	–	–	–	–	9.60	97.0

^(*)^ Description of the study: 75 mL solutions with initial concentrations of 0–1000 mg P/L + 3 g material, shaken for 24 h, ^(**)^ Description of the study: 75 mL solutions with initial concentrations of 5–1000 mg P/L + 3 g material, shaken for 48 h.

**Table 16:** Sorption properties of different varieties of slag.

Material [References]	pH [–]	Initial Phosphorus Concentration [mg P/L]	Sorption Capacity [g P/kg]	Maximum Sorption Capacity [g P/kg]	Phosphorus Removal Efficiency [%]
Furnace slag [68] ^(*)^	12.3	100–1000	–	8.89 ^(L)^	–
Steel slag [70] ^(**)^	–	1–20	1.06–18.88	21.70	98.2
Blast furnace slag [71] ^(***)^	8.5	500	–	6.37 ^(F)^	99.0
CSH from blast furnace slag [72] ^(****)^	12	38–47.5	53.11	75.70 ^(L)^	–
Furnace slag [69] ^(*****)^	11	60	8.80	–	99.0
	11	20	1.64	–	99.0

^(*)^ Description of the study: solutions with initial concentrations of 100–1000 mg P/L + 2 g material, shaken at 200 rpm for 24 h, ^(**)^ Material composition: Fe_2_O_3_—33.92%; SiO_2_—11.81%; MgO—6.98%; CaO—41.09%; description of the study: solutions with initial concentrations of 1–20 mg P/L + 0.04 g material, ^(***)^ Material composition: Fe_2_O_3_—0.33%; Al_2_O_3_—10.82; SiO2—39.56%; MgO—6.79%; CaO—37.68%; description of the study: dose of material 60 g/L + solution with initial concentration of 500 mg P/L, ^(****)^ Material composition: Fe_2_O_3_—1.53%; Al_2_O_3_—14.78%; SiO2—34.58%; MgO—5.29%; CaO—40.09%; description of the study: solutions with initial concentrations of 38–47.5 mg P/L + 0.1–3.5 g material, shaken at 200 rpm for 24 h, ^(*****)^ Material composition: Fe_2_O_3_—21.90%; Al_2_O_3_—7.64%; SiO_2_—12.70%; MgO—9.66%; CaO—38.30%, ^(L)^ Maximum sorption capacity according to the Langmuir model, ^(F)^ Maximum sorption capacity according to the Freundlich model.

**Table 17:** The sorption capacities of modified biochar and coal slag.

References	Material		Composition					Specific Surface Area [m^2^/g]	Sorption Capacity According to the Kinetic Model [g P/kg]	Maximum Sorption Capacity According to the Langmuir Model [g P/kg]
			Fe_2_O_3_ [%]	Al_2_O_3_ [%]	SiO_2_ [%]	CaO [%]	MgO [%]			
[72]	biochar	raw	–	–	–	–	–	61.16	1.80	2.39
		with fly ash	8.36	33.26	41.96	4.88	0.50	80.72	2.11	3.08
		with coal gangue	8.23	31.66	40.80	9.66	0.66	75.86	2.17	3.20
[77]	coal slag		5.14	28.70	55.90	1.04	1.96	9.20	8.85	21.63
[75]	biochar containing MgO nanoparticles		–	–	–	–	–	273.82	–	60.95
[76]	dolomite—modified biochar		–	–	–	–	–	11.31	20.48	29.18

**Table 18:** Analysed sorption properties of autoclaved concrete.

References	Fraction [mm]	Initial Phosphorus Concentration [mg P/L]	Maximum Sorption Capacity According to the Langmuir Model [g P/kg]	Phosphorus Removal Efficiency [%]	Final pH [–]
[80] ^(*)^	2.0–4.0	10.0	70.90	100.0	8.5–9.3
[82] ^(**)^	2.0–4.0	100.0	14.29	–	–
[83] ^(***)^	0.125–0.250	0.2–0.3	0.28	65.2–86.7	10.3–11.3
[81] ^(****)^	dust	25.0	7.93	94.0	12.3
[84] ^(*****)^	2.0–5.0	4.9–1108.7	9.00	–	–

^(*)^ Material composition: Fe_2_O_3_—1.10%; SiO_2_—51.40%; CaO—26.30%; Al_2_O_3_—1.95%. Phosphorus removal efficiency of 100% for an initial concentration of 5 mg P/L, ^(**)^ Description of the study: solution with initial concentration of 100 mg P/L + material, shaken at 150 rpm for 4h, ^(***)^ Description of the study: solutions with initial concentrations of 0.2–0.3 mg P/L + material, shaken for 1 h, ^(****)^ Material composition: Fe_2_O_3_—3.06%; SiO_2_—20.88%; CaO—53.50%; Al_2_O_3_—6.13%; MgO—2.39; description of the study: material + solutions with initial concentrations of 25 mg P/L, shaken at 150 rpm for 8 h, ^(*****)^ Material composition: Fe_2_O_3_—1.03%; SiO_2_—57.24%; CaO—24.60%; Al_2_O_3_—1.96%; MgO—0.52; description of the study: material + solutions with initial concentrations of 4.87–1108.7 mg P/L, shaken for 0.5 h.

**Table 19:** Synthesized materials.

Material	Specific Surface Area [m^2^/g]	Maximum Sorption Capacity According to the Langmuir Model [g P/kg]
Magnetic diatomite nanocomposite (MDC)	–	11.89
Illite clay nanocomposite (MIC)	–	5.48
Modified montmorillonite AlPMt	283.20	10.07–13.04
Nano-valent iron barriers (nZVI)	43.09	54.34
Goethite synthesized	100.00	18.20–27.00

**Table 20:** Comparison of powdered ceramsite with brick dust.

References	Description of the Study	Material	Composition					Specific Surface Area [m^2^/g]	Maximum Sorption Capacity According to the Langmuir Model [g P/kg]
			Fe_2_O_3_ [%]	Al_2_O_3_ [%]	SiO_2_ [%]	CaO [%]	MgO [%]		
[93]	50 mL solutions with initial concentrations of 2–200 mg P/L + material, shaken (24 h)	Powdered ceramsite	12.91	17.56	46.28	10.62	3.58	5.18	0.59
		brick dust	7.29	18.83	62.33	2.14	2.24	4.86	0.46

**Table 21:** Properties of industrial waste materials.

Description of the Study	Material	Composition CaO [%] Fe_2_O_3_ [%] Al_2_O_3_ [%]			Initial pH [–]	Final pH [–]	Sorption Capacity [g P/kg]
solutions with initial concentrations 0–103.23 mg P/L + 2 g material, contact time: 16 h, centrifugation: 2000 rpm for 15 min	ADMR1	30.10	8.81	0.16	8.2	7.21	40.0
	ADMR2	2.26	10.28	5.55	9.1	8.30	29.0
	WTR	0.52	3.16	7.64	7.5	6.57	32.0
	FGD	19.84	0.13	0.05	7.9	4.46	14.0
	Red mud	3.86	1.20	2.65	8.2	6.66	29.0
	Fly ash	1.27	5.56	7.78	9.8	7.90	25.0

**Table 22:** Results for red mud and fly ash.

Material		Composition					Specific Surface Area [m^2^/g]	Phosphorus Removal Efficiency [%]	Maximum Sorption Capacity According to the Langmuir Model [g P/kg]
		Fe_2_O_3_ [%]	Al_2_O_3_ [%]	SiO_2_ [%]	CaO [%]	MgO [%]			
Red mud	raw	12.76	6.93	19.14	46.02	1.15	14.09	47.8	113.87
	activated 0.25HCl	14.84	7.20	20.34	45.16	1.42	19.35	99.0	161.61
	calcinated at 700 °C	13.05	8.06	22.45	45.23	1.14	9.69	99.0	345.50
Fly ash	raw	7.35	25.36	56.38	2.72	1.45	14.55	16.1	63.22
	activated 0.25HCl	7.02	27.10	56.75	2.13	1.85	18.70	45.2	78.44
	calcinated at 700 °C	6.09	28.47	57.2	2.14	1.57	12.20	52.9	58.92

**Table 23:** Results of research on iron oxide tailings and clinker ash.

Material	Composition					Physical Parameters				Maximum Sorption Capacity According to the Langmuir Model
	Fe_2_O_3_ [%]	Al_2_O_3_ [%]	SiO_2_ [%]	CaO [%]	MgO [%]	Bulk Density	Particle Density	Porosity	Hydraulic Conductivity	
						[g/m^3^ ]	[g/m^3^]	[%]	[m/s]	[g P/kg]
Iron oxide tailings	25.10	5.40	18.50	4.20	3.00	1.00	2.00	48.50	0.00011	1.29
Clinker ash	5.30	11.70	18.60	6.00	0.40	1.30	2.90	56.70	2.60	0.29

**Table 24:** Waste from the agri-food industry.

Material	Maximum Sorption Capacity According to the Langmuir Model [g P/kg]	Initial pH [–]	Final PH [–]	Optimal pH [–]	Final pH of the Solution at Optimal pH [–]
*Arachis hypogaea* husks, unmodified	0.01	5.00–8.00	6.94–7.54	8.00	7.54
*Arachis hypogaea* husks, modified	0.10	5.00–8.00	6.64–7.85	5.00	6.64
Chitin	2.09	5.00–7.00	6.74–7.10	3.00	3.33
Chitosan	6.64	4.00–8.00	6.75–7.86	4.00	6.75

**Table 25:** Man–made materials.

References	Material	Description of the Study	pH [–]	Porosity [%]	Sorption Capacity [g P/kg]
[92]	*Polonite* ^®^	solutions with initial concentrations of 100–1100 mg P/L + 1 g material, shaken for 24 h	12.0	38.00	40.90 ^(L)^
[92]	*Leca* ^®^		7.5	48.00	5.10 ^(L)^
[105]	LWA	solutions with initial concentrations of 0–320 mg P/L + 8 g material, shaken for 24 h	–	–	3.47
[106]	LWA	solutions with initial concentrations of 320–480 mg P/L + 3 g material, shaken for 24 h	–	–	12.00
[107]	*Rockfos* ^®^^(*)^	solutions with initial concentrations of 5–100 mg P/L + 10 g material shaken for 1 h	11.0–12.0	>50.00	256.40 ^(L)^
[108]	*Pollytag* ^®^^(**)^	solutions with initial concentrations of 1.28–949 mg P/L + 1 g material, shaken for 15 min	7.4	62.00	32.24
[64]	*Filtralite* ^®^	do solutions with initial concentrations of 0–480 mg P/L + 3 g material, shaken for 24 h	10.7	68.00	2.50 ^(L)^
[110]	*Filtra P* ^(***)^	do solutions with initial concentrations of 3–1000 mg P/L + 25 g material, shaken for 48 h	–	–	4.30

^(*)^ Material composition: Fe_2_O_3_—1.34%; Al_2_O_3_—5.93; CaO—43.34%, ^(**)^ Material composition: Fe_2_O_3_—7.50%; Al_2_O_3_—24.30; CaO—4.50%, ^(***)^ Material composition: Fe_2_O_3_—0.46%; Al_2_O_3_—0.86; CaO—49.70%, ^(L)^ Maximum sorption capacity according to the Langmuir model.
